# Microbiological findings of dental implants among irradiated head and neck cancer patients

**DOI:** 10.6026/973206300212383

**Published:** 2025-08-31

**Authors:** Himanshu Sharma, Surabhi Duggal, Sahba Hassan, Lalit Kumar, Shailesh Jain, Maitreyi Ranjan

**Affiliations:** 1 Department of Oral & Maxillofacial Surgery, Maharishi Markandeshwar College of Dental Sciences and Research, Maharishi Markandeshwar (Deemed to be University) Mullana, Ambala, Haryana, India; 2 Department of Prosthodontics and Crown & Bridge, School of Dental Sciences, Sharda University, Greater Noida, Uttar Pradesh, India; 3 Department of Prosthodontics and Crown & Bridge, DJ College Of Dental Sciences and Research, Modinagar, Uttar Pradesh, India; 4 Prosthodontics, Medical Officer (Dental) CHC Chandi, District Solan, Himachal Pradesh, India; 5 Department of Prosthodontics and Crown & Bridge, Bhojia Dental College, Baddi, Himachal Pradesh, India; 6 Department of Microbiology, School of Dental Sciences, Sharda University, Greater Noida, Uttar Pradesh, India

**Keywords:** Radiotherapy, dental implant, head and neck cancer, microbiological environment

## Abstract

The microbiological profile surrounding healthy osseointegrated dental implants in patients treated with radiotherapy for head and neck cancer. Radiation therapy is known to alter the oral environment, potentially affecting peri-implant microbial colonization. Samples from irradiated patients with clinically healthy implants were analyzed for microbial composition. Thus, we show a distinct microbial signature compared to non-irradiated individuals, though without signs of peri-implant disease. These results highlight the importance of tailored monitoring protocols in post-radiotherapy implant care.

## Background:

With over 800,000 new cases diagnosed each year and a high death rate, particularly in low- and middle-income nations, head and neck cancers (HNCs) pose a serious threat to global health [1]. Although radiotherapy is still a mainstay in the treatment of HNCs, it frequently results in long-term oral side effects like xerostomia, mucositis, and impaired bone healing [2]. Dental implant placement and survival in irradiated jawbones may be adversely affected by these complications [3]. Dental implants have transformed oral rehabilitation for cancer survivors; however, because of altered bone metabolism and vascularity, implant placement in irradiated sites poses special challenges [4]. Concerns about peri-implant health and microbial colonisation in this population remain, despite the fact that multiple studies show acceptable implant survival rates in irradiated patients [5, 6]. The oral microbiota is essential for preserving the health of the tissue surrounding implants. Microbial imbalance, or dysbiosis, may make irradiated patients more susceptible to peri-implant disease [7]. To increase long-term implant success, it is essential to comprehend the microbial changes in these patients. Although results are still inconclusive, previous studies have demonstrated changed microbial diversity and an increase in opportunistic pathogens in irradiated individuals [8]. In oral rehabilitation, the implantation of dental implants in irradiated jawbones is still a contentious but growingly studied topic. Although radiotherapy is crucial for treating head and neck cancers, it impairs the jawbones' vascularity and ability to heal, which raises questions regarding implant survival and the possibility of side effects like osteoradionecrosis (ORN) [9]. Although irradiated patients have lower implant survival rates than non-irradiated patients, systematic reviews have shown that these rates are still within clinically acceptable bounds, especially when adjunctive measures like delayed loading or hyperbaric oxygen therapy are used [10]. But it's important to understand that microbial imbalance, poor hygiene, and a changed immune response after radiation can also contribute to implant failure in these patients, underscoring the necessity of thorough assessment and monitoring [11]. Using both culture techniques and 16S rRNA sequencing, Therefore, it is of interest to evaluate the microbial diversity, community composition, and clinical stability of dental implants in irradiated HNC patients, with a comparison to healthy controls.

## Methodology:

## Study design and population:

This was a cross-sectional observational study conducted on head and neck cancer patients who had received radiotherapy and subsequently underwent dental implant placement. Participants were recruited from the oral oncology and prosthodontics departments of a tertiary care center. Inclusion criteria comprised patients aged ≥18 years, with a history of completed head and neck radiotherapy (minimum 6 months post-treatment) and clinically healthy peri-implant tissues. Exclusion criteria included active peri-implant disease, systemic antibiotic use within 3 months, or uncontrolled systemic illnesses (*e.g.*, diabetes mellitus, immunosuppression).

## Clinical examination:

A thorough peri-implant examination was performed, including probing depth (PD), bleeding on probing (BOP) and radiographic assessment. Only implants with no signs of inflammation or bone loss were included.

## Microbiological sampling:

Subgingival plaque samples were collected from the peri-implant sulcus using sterile paper points, which were inserted for 30 seconds and immediately transferred to transport media. Samples were processed using culture-based methods and 16S rRNA sequencing to identify bacterial taxa. Colony-forming units (CFU) were counted for quantitative assessment.

## Microbial identification:

DNA was extracted using a commercial extraction kit and 16S rRNA gene amplification was performed targeting the V3-V4 regions. Sequencing was done using an IlluminaMiSeq platform. Bioinformatics analysis was conducted using QIIME 2 software to determine alpha and beta diversity and taxonomic classification was carried out using the SILVA database.

## Statistical analysis:

Data were analyzed using SPSS Version 26.0 (IBM Corp., Armonk, NY). Descriptive statistics (mean, standard deviation, median, interquartile range) were used to summarize demographic and clinical variables.

## Microbial diversity:

Alpha diversity indices (Shannon, Simpson) were calculated to assess species richness and evenness. Differences were compared using the Kruskal-Wallis test or Mann-Whitney U test.

## Taxonomic comparison:

The relative abundance of bacterial genera between irradiated and non-irradiated groups (if applicable) was compared using ANOVA or Wilcoxon rank-sum tests.

## Multivariate analysis:

Principal Coordinate Analysis (PCoA) and PERMANOVA were used to assess clustering of microbial communities. A p-value of <0.05 was considered statistically significant.

## Results:

A total of 30 irradiated head and neck cancer patients (18 males, 12 females; mean age: 59.3 ± 8.4 years) with 45 clinically healthy dental implants were included. The mean duration since radiotherapy was 18.2 ± 6.7 months. All implants demonstrated successful osseointegration with no signs of peri-implant inflammation or bone loss. Clinical parameters such as probing depth (2.4 ± 0.5 mm), bleeding on probing (6.7%) and marginal bone loss (0.2 ± 0.1 mm) indicated stable peri-implant health (Table 2 - see PDF). Microbiological profiling of the peri-implant sulcus in irradiated patients revealed a distinctive bacterial composition. The most commonly identified genera were *Streptococcus* (32.4%), *Actinomyces* (18.1%), *Lactobacillus* (11.3%) and *Prevotella* (9.6%), with *Candida albicans* detected in 26.7% of samples through culture methods (Table 1 - see PDF). Although opportunistic fungi like *C. albicans* were present, they were not associated with clinical signs of infection. Alpha diversity indices showed significantly reduced microbial richness and evenness in irradiated patients compared to healthy controls. The Shannon Index was significantly lower in the irradiated group (2.91 ± 0.48) than in the control group (3.65 ± 0.52, p < 0.01), indicating decreased diversity (Table 3 - see PDF). Other indices, such as Observed Species, Chao1 Richness, and Simpson Index, also demonstrated statistically significant reductions in the irradiated group (p < 0.01). Furthermore, Principal Coordinates Analysis (PCoA) revealed distinct clustering of microbial community structures between irradiated and control groups, supporting the hypothesis that radiation therapy significantly alters the peri-implant microbiome (Figure 1 - see PDF).

## Discussion:

The current results support the idea that, even in clinically healthy implants, radiation-induced changes in the oral environment have a major effect on the peri-implant microbiota. Decreased microbial richness and evenness are reflected in reduced alpha diversity seen in irradiated patients. This pattern has also been reported in studies evaluating early and late implant failures, where microbial imbalance was connected to implant loss [12]. Notably, our study found opportunistic pathogens like *Candida albicans* even in the absence of a clinical infection, underscoring the subclinical alterations in mucosal immunity and local microbiota dynamics that radiation can induce [13]. Peri-implantitis is not only caused by traditional periodontal pathogens, but is also impacted by the larger microbial community and its ecological changes, according to next-generation sequencing (NGS) research [14]. The oral microbiome's resilience is weakened in irradiated people, which reduces its ability to bounce back from disturbances like pH shifts, salivary flow, or mechanical trauma [17]. The different microbial clustering between the irradiated and control groups in our study may be explained by this. Furthermore, maintaining oral health and function is part of cancer care support that goes beyond oncologic treatment, especially for patients who depend on implants for prosthetic rehabilitation [15]. Patients may be at risk for dysbiosis, which can trigger inflammatory pathways even in the absence of clinical peri-implantitis, due to the long-term immunosuppression and tissue damage brought on by radiation therapy [14]. As observed in other cancers where non-coding RNAs regulate cellular migration and repair mechanisms, such immune and microbial reactions may also interact with systemic pathways [16]. Additionally, prior research has shown that irradiated bone offers a distinct healing environment with changed bone metabolism and vascularity, which may not show up right away but may have an impact on long-term microbial-host interactions surrounding implants [19]. This backs up the increasing focus on individualised maintenance plans and routine microbial surveillance in irradiated patients. There is also a new perspective that suggests a possible connection worth investigating further in irradiated patients: systemic skeletal bone density is correlated with periodontitis and peri-implant conditions [20]. Furthermore, the technical and analytical difficulties in precisely characterising these communities have been brought to light by sophisticated methods in oral microbiome research, particularly in biopsies related to cancer [21]. However, it is impossible to overstate the importance of comprehending microbial changes in these kinds of environments. The importance of cautious antibiotic stewardship and awareness of emerging pathogens in vulnerable populations, such as cancer survivors with implants, is further highlighted by genetic and microbial resistance factors that have been identified in broader microbiological contexts (*e.g.*, carbapenem-resistant *E. coli*) [18].

## Conclusion:

There is a presence of distinct microbial signature compared to non-irradiated individuals, though without signs of peri-implant disease. Longitudinal studies with larger sample sizes and functional metagenomics could provide deeper insights into host-microbe interactions in the peri-implant environment.
